# The role of obesity in the immune response during sepsis

**DOI:** 10.1038/nutd.2014.34

**Published:** 2014-09-22

**Authors:** A S Kolyva, V Zolota, D Mpatsoulis, G Skroubis, E E Solomou, I G Habeos, S F Assimakopoulos, N Goutzourelas, D Kouretas, C A Gogos

**Affiliations:** 1 Division of Infectious Diseases, Department of Internal Medicine, Patras University Hospital, Rion-Patras, Greece; 2 Department of Pathology, Patras University Hospital, Rion-Patras, Greece; 3 Department of Surgery, Patras University Hospital, Rion-Patras, Greece; 4 Department of Internal Medicine, Patras University Hospital, Rion-Patras, Greece; 5 Division of Endocrinology, Department of Internal Medicine, Patras University Hospital, Rion-Patras, Greece; 6 Department of Biochemistry and Biotechnology, University of Thessaly, Larissa, Greece

## Abstract

**Background/Objectives:**

Sepsis is one of the most important causes of mortality in the developed world, where almost two-thirds of the population suffer from obesity. Therefore, the coexistence of both conditions has become frequent in clinical practice and a growing number of clinical studies attempts to examine the potential effect of obesity on sepsis with controversial results up to now. The present study investigates how obesity influences the immune response of septic patients, by assessing the number and activation state of adipose tissue macrophages, serum and adipose tissue tumor necrosis factor-alpha (TNFα) levels and plasma oxidative stress markers.

**Subjects/methods:**

The study included 106 patients, divided into four groups (control *n*=26, obesity *n*=27, sepsis *n*=27 and sepsis and obesity *n*=26). The number of macrophages in subcutaneous and visceral adipose tissue (SAT and VAT) and their subtypes (M1 and M2) were defined with immunohistochemical staining techniques under light microscopy. TNFα mRNA levels were determined in SAT and VAT using real-time reverse transcription-PCR. Serum levels of TNFα were determined with sandwich enzyme-linked immunosorbent assay. Plasma oxidative stress was evaluated using selective biomarkers (thiobarbituric acid-reactive substances (TBARS), protein carbonyls and total antioxidant capacity (TAC)).

**Results:**

Sepsis increased the total number of macrophages and their M2 subtype in (VAT), whereas obesity did not seem to affect the concentration of macrophages in fat. Obesity increased TNFα mRNA levels (*P*<0.05) in VAT as well as the plasma TBARS (*P*<0.001) and protein carbonyls (*P*<0.001) in septic patients. The plasma TAC levels were decreased and the serum TNFα levels were increased in sepsis although they were not influenced by obesity.

**Conclusions:**

Obesity is associated with elevated TNFα adipose tissue production and increased oxidative stress biomarkers, promoting the proinflammatory response in septic patients.

## Introduction

Sepsis is defined as the systemic inflammatory response syndrome attributed to infection. It is the leading cause of morbidity and mortality in noncardiac intensive care units worldwide and it is related with a high cost of care.^1,2^ Pathophysiologically, it is characterized by the activation of endothelial cells and monocytes and the initiation of inflammatory and coagulation cascades, in response to invading pathogens leading to collateral damage of normal tissues.^3^ The severity and outcome of the septic syndrome depend on the original site and type of infection, the host's response to infection and the time of institution of appropriate antimicrobial therapy.^4, 5, 6, 7^

Obesity, a chronic metabolic disease characterized by excessive fat accumulation and overgrowth of adipose tissue, has reached epidemic proportions over the past few decades leading to substantial morbidity and mortality.^8, 9, 10, 11, 12^ The interaction between obesity and infectious diseases has been increasingly investigated, particularly after the emerging data indicating an association between obesity and poor outcome in the pandemic H1N1 influenza infection.^13,14^ Currently, obesity is considered as an established risk factor for pancreatitis, surgical-site, nosocomial and skin infections.^15^ On this basis, the potential negative impact of obesity on the outcome of septic patients is an area of growing research interest over the past years; however, no conclusive evidence exists on this issue and several pathophysiological gaps remain to be filled.

Host's immune response and its dysregulation in sepsis is majorly dependent on the expression and secretion of a variety of pro- and anti-inflammatory cytokines, which additionally display pivotal role in the obesity-associated chronic inflammatory activation and its subsequent metabolic abnormalities such as insulin resistance.^16, 17, 18, 19^ Tumor necrosis factor-alpha (TNFα) is a representative proinflammatory cytokine with a prominent role in the inflammatory response of sepsis and obesity.^16, 17, 18^ According to previous studies, this inflammatory response is at least partly promoted by cytokines produced by adipose tissue macrophages, which undergo quantitative and qualitative alterations in obesity and sepsis.^20, 21, 22, 23, 24^ Adipose tissue macrophages are subdivided in two major populations: the classically activated M1 macrophages that produce proinflammatory cytokines and the alternatively activated M2 macrophages that secrete anti-inflammatory cytokines.^25,26^ Another important pathophysiological factor associated with the injurious effects of sepsis and obesity on various organs' structure and function is oxidative stress, driven by the imbalance of tissue oxidants (free radicals or reactive oxygen species) and antioxidants defenses in favor of the former.^27, 28, 29, 30, 31^

The present study was undertaken to investigate the effect of obesity in the immunologic response in sepsis, by studying the potential alterations in: (a) the adipose tissue macrophages and TNFα production; and (b) the systemic inflammatory response as assessed by serum TNFα and plasma oxidative stress.

## Patients and methods

In this prospective study, a total of 106 patients (51 females and 55 males), who were admitted to Patras University General Hospital in Greece during a 5-year period (October 2008 to May 2013) were enrolled. According to our protocol, the patients were divided into four groups. The inclusion criteria of each group are described below:

1. Control group (*n*=26) included individuals with body mass index (BMI) <30 without clinicolaboratory signs of infection. In addition to the blood sample, in 11 out of 26 patients, samples of visceral adipose tissue (VAT) and subcutaneous adipose tissue (SAT) were obtained as they underwent scheduled laparoscopic surgery for noninfectious and nonneoplastic etiologies (cholecystectomy (*n*=8), hernia repair surgery (*n*=2) and ovarian cyst removal surgery (*n*=1)). The rest of the patients (*n*=15) were admitted to the hospital due to scheduled cataract surgery.
2. Obesity group (*n*=27) included individuals with BMI ⩾30 without clinicolaboratory signs of infection. In addition to the blood sample, in 11 out of 27 patients, samples of VAT and SAT were obtained as they underwent scheduled laparoscopic surgery for noninfectious and nonneoplastic etiologies (cholecystectomy (*n*=5) and bariatric surgery (mini-gastric bypass, *n*=6)). The rest of the patients (*n*=16) were admitted to the hospital owing to scheduled cataract surgery.
3. Sepsis group (*n*=27) included individuals with BMI <30 with sepsis. In addition to the blood sample, in 11 out of 27 patients, samples of VAT and SAT were obtained as they underwent urgent abdominal operation for localized intra-abdominal infection (acute cholecystitis (*n*=6) or acute appendicitis (*n*=5) without peritonitis—clinically diagnosed and confirmed by pathology). The rest of the patients (*n*=16) were admitted to the hospital with lower respiratory tract infection, acute febrile gastroenteritis or upper urinary tract infection.
4. Sepsis & obesity group (*n*=26) included individuals with BMI ⩾30 with sepsis. In addition to the blood sample, in 11 out of 26 patients, samples of VAT and SAT were obtained as they underwent urgent abdominal operation for localized intra-abdominal infection (acute cholecystitis (*n*=4) or acute appendicitis (*n*=7) without peritonitis—clinically diagnosed and confirmed by pathology). The rest of the patients (*n*=15) were admitted to the hospital with lower respiratory tract infection, acute febrile gastroenteritis or upper urinary tract infection.

The characteristics of the study's population are presented in Table 1. There were no significant differences considering the number and the age of the patients enrolled in the four study groups. The BMI of the nonobese groups (control group and sepsis group) was significantly different from the BMI of the obese groups (obese group and sepsis & obese group) (*P*<0.001).

Sepsis was defined according to the criteria of the American College of Chest Physicians—Society of Critical Care Medicine Consensus Conference Committee—as the presence of confirmed infection and ⩾2 of the following criteria: (a) a temperature of >38 °C or <36 °C; (b) a heart rate of ⩾90 beats per min; (c) tachypnea, manifested by a respiratory rate of ⩾20 breaths per min or hyperventilation, indicated by a PaCO_2_ of <32 mm Hg; and (d) an altered white blood cell count of>12 000 or <4000 cells per mm^3^ or the presence of >10% immature forms.

Obesity was defined according to World Health Organization (2004) ‘International Classification of adult underweight, overweight and obesity' by BMI, so that patients with BMI ⩾30 kg m^−2^ were considered obese.

Criteria for exclusion were the presence of severe comorbidities (malignancy, chronic liver or renal disease, chronic obstructive pulmonary disease, heart failure, diabetes mellitus, rheumatic diseases under immunosuppressive therapy, uncontrolled endocrine disease, HIV infection, hypogammaglobulinemia or other primary immunodeficiency), gastrointestinal diseases (celiac disease, inflammatory bowel disease) and current treatment with antiobesity medications, corticosteroids, nonsteroid anti-inflammatory drugs and antioxidants (vitamins C and E, allopurinol and *N*-acetyl-cysteine).

Diagnostic procedures (blood tests, chest X-rays, ultrasound, computed tomography and so on) were performed upon admission and during hospitalization to identify the source of infection. Body weight and height were measured (or self-reported if measurement was not possible) at admission, for calculation of BMI.

The study was approved by Patras University General Hospital Ethics Committee. The use of human material conforms to the principles outlined in the Declaration of Helsinki.

### Sampling and assays

#### Number of macrophages in adipose tissue

SAT (from the anterior abdominal wall) and VAT samples were obtained during surgery. Care was taken to sample VAT away from the gross inflammatory site in patients with intra-abdominal sepsis. One part of these samples was submerged immediately after collection in buffered formalin solution for 24 h, grossly sliced, paraffin embedded and then sectioned (thin sections, 4 μm thick). Sections were processed for immunohistochemical detection of macrophage marker CD68 (clone PG-M1, monoclonal mouse anti-human, Dako, Glostrup, Denmark) and macrophage subtype M2 and M1 markers, CD206 (monoclonal mouse anti-human, AbD Serotec, Hsi-Chih, Taiwan) and CD80 (monoclonal rabbit anti-human, Abcam, Shanghai, China), respectively, according to the protocol of Zolota *et al.*^32^

The total macrophages (CD68+) as well as the M2 (CD206+) and M1 (CD80+) macrophage subtypes were systematically counted on each processed slide under light microscopic examination in 10 high-power fields (at × 40 magnification), and their mean number was evaluated excluding macrophage-poor areas. The number of macrophages was normalized to 100 adipocytes for comparison between patients.

#### RNA isolation and expression of TNFα mRNA in adipose tissue

The second part of the SAT and VAT samples obtained during surgery was submerged immediately after collection in RNA stabilization and storage solution (RNAlater Solution, Ambion, Foster City, CA, USA), left at room temperature for 24 h and stored in eppendorf tubes at −70 °C until analysis.

Total RNA was isolated using TRIzol reagent (Invitrogen, Paisley, UK) and RNeasy Mini kit (Qiagen, Valencia, CA, USA) for the purification of the RNA. Standard procedures were followed according to the manufacturers' instructions. A DNAse I digestion step was included to prevent genomic DNA contamination (Invitrogen). The quality of total RNA was assessed using the Agilent 2100 Bioanalyzer (Agilent Technologies, Santa Clara, CA, USA), following the Agilent RNA 6000 Nano Assay Protocol. Total RNA was converted to complementary DNA by reverse transcription using the Superscript first-strand synthesis system (Invitrogen). The complementary DNA was amplified by real-time PCR using the Step One Plus instrument (Applied Biosystems, Foster City, CA, USA). TaqMan Gene Expression Master Mix, Taqman gene expression assay for human TNFα Hs00174128_m1 (4331182) 20 × and Taqman gene expression assay for human cyclophilin A (4333763F) 20 × (Invitrogen) were used according to the following protocol: 40cycles (10 s at 95 °C and 1 min at 60 °C). All reactions were performed in triplicates. Relative expression of TNFα mRNA among the four groups was performed using the ΔΔCt method.

#### Serum cytokines

Within 24 h after patients' admission and preoperatively for surgical patients, serum for cytokine determination was obtained from whole blood by centrifugation (4000 *g* for 5 min). Samples were subsequently stored in multiple aliquots at −30 °C until analysis. Serum samples were thawed only once before analysis and each assay was performed in duplicates. Determination of TNFα levels was performed by quantitative sandwich enzyme-linked immunosorbent assay using Human TNFα/TNFSF1A (Quantikine R&D Systems, Inc., Minneapolis, MN, USA). Standard procedures were followed according to the manufacturer's instructions. The absorbance was read at 450 nm (Tecan, Sunrise ELISA reader, Grödig, Austria). To convert optical density to concentration (pg ml^−1^), a standard curve was created using computer software (Excel 2007, Microsoft Corporation, Redmond, WA, USA) for generating a four-parameter logistic curve fit.

#### Oxidative stress and antioxidant capacity in plasma

Within 24 h after patients' admission and preoperatively for surgical patients, plasma for oxidative stress and antioxidant capacity evaluation was obtained from whole blood by centrifugation (4000 *g* for 5 min). Samples were subsequently stored in multiple aliquots at −30 °C until analysis.

The thiobarbituric acid-reactive substances (TBARS) test assesses lipid peroxidation, which results in the formation of malondialdehyde. For the concentration of TBARS, a slightly modified assay of Keles *et al.*^33^ was used. According to this method, 100 μl of plasma was mixed with 500 μl of 35% trichloroacetic acid (Merck, Darmstadt, Germany) and 500 μl of Tris–HCl (Sigma-Aldrich, St Louis, MO, USA) (200 mM, pH 7.4) and incubated for 10 min at room temperature. One milliliter of 2M Na_2_SO_4_ (Sigma-Aldrich, Steinhain, Germany) and 55 mM thiobarbituric acid solution (Sigma-Aldrich, Steinhain, Germany) were added and the samples were incubated at 95 °C for 45 min. The samples were cooled with ice for 5 min, vortexed after adding 1 ml of 70% trichloroacetic acid and centrifuged at 11 200 *g* for 3 min. The final product was the malondialdehyde (thiobarbituric acid)_2_ adduct, whose absorbance was measured at 530 nm. A baseline shift in absorbance was taken into account by running a blank along with all samples during the measurement.

The concentration of protein carbonyls, an index of protein oxidation, was determined based on the method of Patsoukis *et al.*^34^

The determination of total antioxidant capacity (TAC) was based on the method of Janaszewska and Bartosz.^35^

### Statistical analysis

Data are expressed as the mean±s.d. and in case of non-Gaussian distribution as the median and 25–75% percentile. One-way analysis of variance (followed by Turkey's multiple comparison test or its nonparametric equivalent, the Kruskal–Wallis test) were performed using GraphPad Prism 5 (GraphPad Software, La Jolla, CA, USA). *P*<0.05 was considered significant.

## Results

### Adipose tissue macrophages

**Figure 1:**
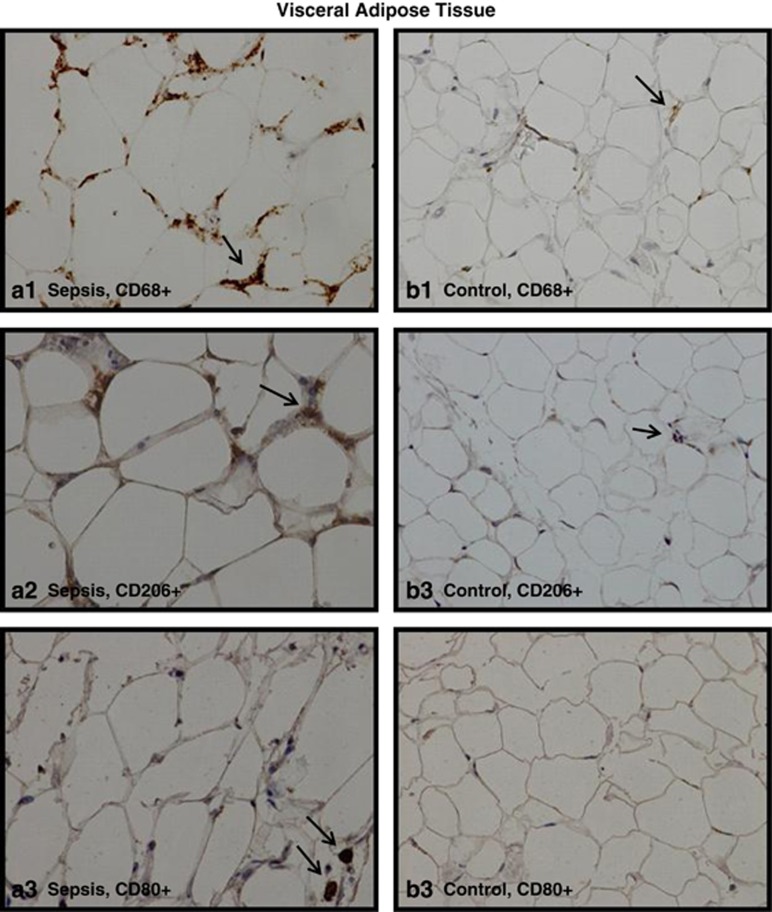
Representative microscopic photographs ( × 40 magnification) for detection and quantification of CD68+, CD206+ and CD80+ cells in human adipose tissue. Immunopositivity for CD68 (images **a1** and **b1**—total macrophages), CD206 (images **a2** and **b2**—M2 macrophages) and CD80 (images **a3** and **b3**—M1 macrophages) in the VAT of a septic patient (**a1**, **a2** and **a3**) and a patient from the control group (**b1**, **b2** and **b3**).

**Figure 2:**
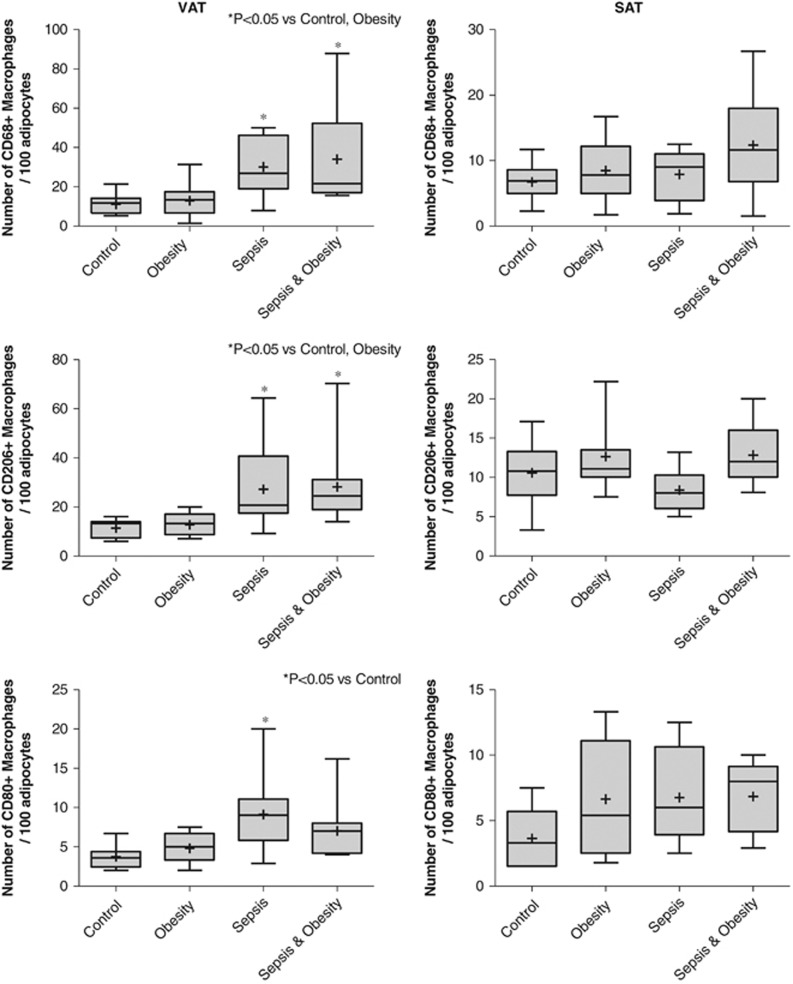
Number of CD68+, CD206+ or CD80+ macrophages per 100 adipocytes in the VAT and SAT of the four study groups (controls *n*=11, obesity *n*=11, sepsis *n*=11 and sepsis & obesity *n*=11).

In all study groups, single CD68+, CD206+ or CD80+ cells were dispersed throughout the parenchyma in VAT and SAT, whereas ‘crown'-like arrangements of macrophages around a single adipocyte were occasionally observed (Figure 1). The number of total macrophages and the number of M2 and the M1 subtypes per 100 adipocytes in VAT and SAT of the four study groups are shown in Figure 2.

In VAT, the density of CD68+ and CD206+ macrophages was statistically significantly higher in all septic patients (obese and nonobese) compared with nonseptic patients (obese and controls) (*P*<0.05). No difference was found for both parameters between control and obesity groups as well as between sepsis and sepsis & obesity groups. The number of CD80+ macrophages was significantly higher in the sepsis group compared with the control group (*P*<0.01) (Figure 2).

In SAT, there were no statistically significant differences in the number of CD68+, CD206+ or CD80+ cells per 100 adipocytes among the four study groups (Figure 2).

### TNFα in adipose tissue

**Figure 3:**
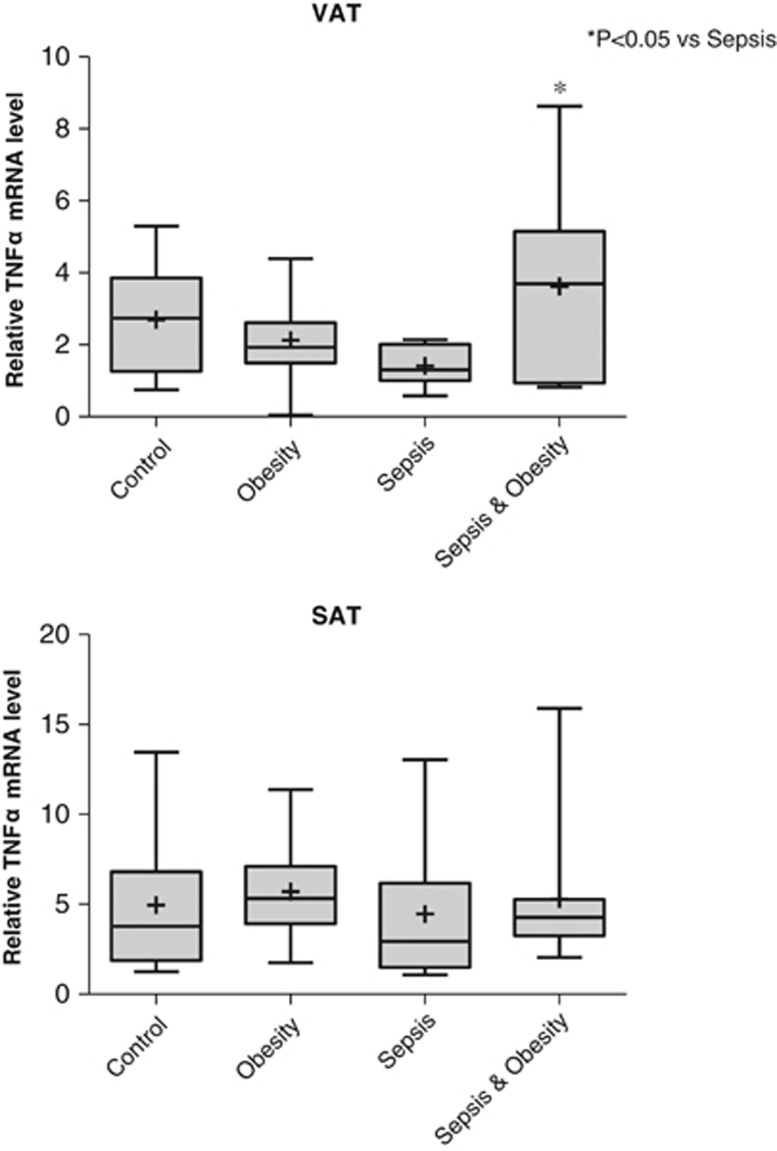
Relative TNFα mRNA levels of the four study groups (controls *n*=10, obesity *n*=11, sepsis *n*=11 and sepsis & obesity *n*=11) in VAT and SAT.

The relative TNFα mRNA levels in VAT and SAT in the four study groups are shown in Figure 3. In VAT, TNFα mRNA levels were statistically significantly higher in the sepsis & obesity group compared with the sepsis group (*P*<0.05). In SAT, there were no statistically significant differences in TNFα mRNA levels among the four study groups.

### Serum TNFα

**Figure 4:**
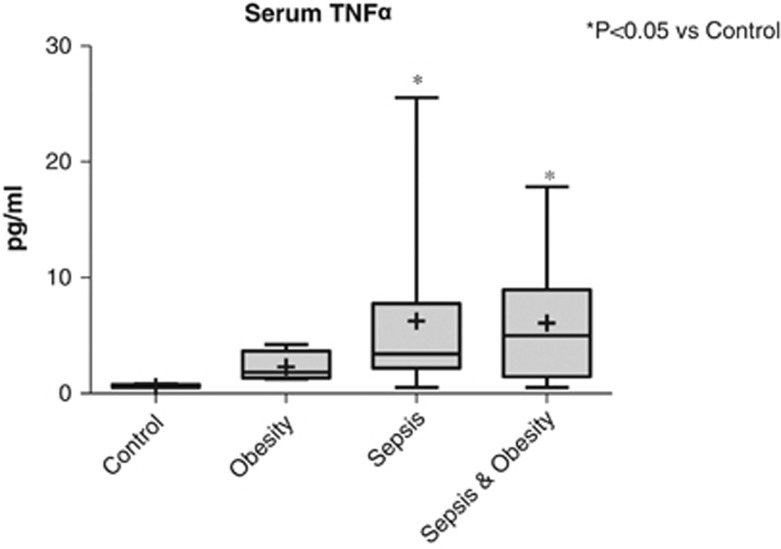
TNFα serum levels of the four study groups (controls *n*=9, obesity *n*=9, sepsis *n*=11 and sepsis & obesity *n*=11).

The serum levels of TNFα in the four study groups are shown in Figure 4. The serum TNFα levels were statistically significantly higher in all septic patients (obese and nonobese) compared with the control group (*P*<0.05). No difference between sepsis and sepsis & obesity groups was detected.

### Oxidative stress

**Figure 5:**
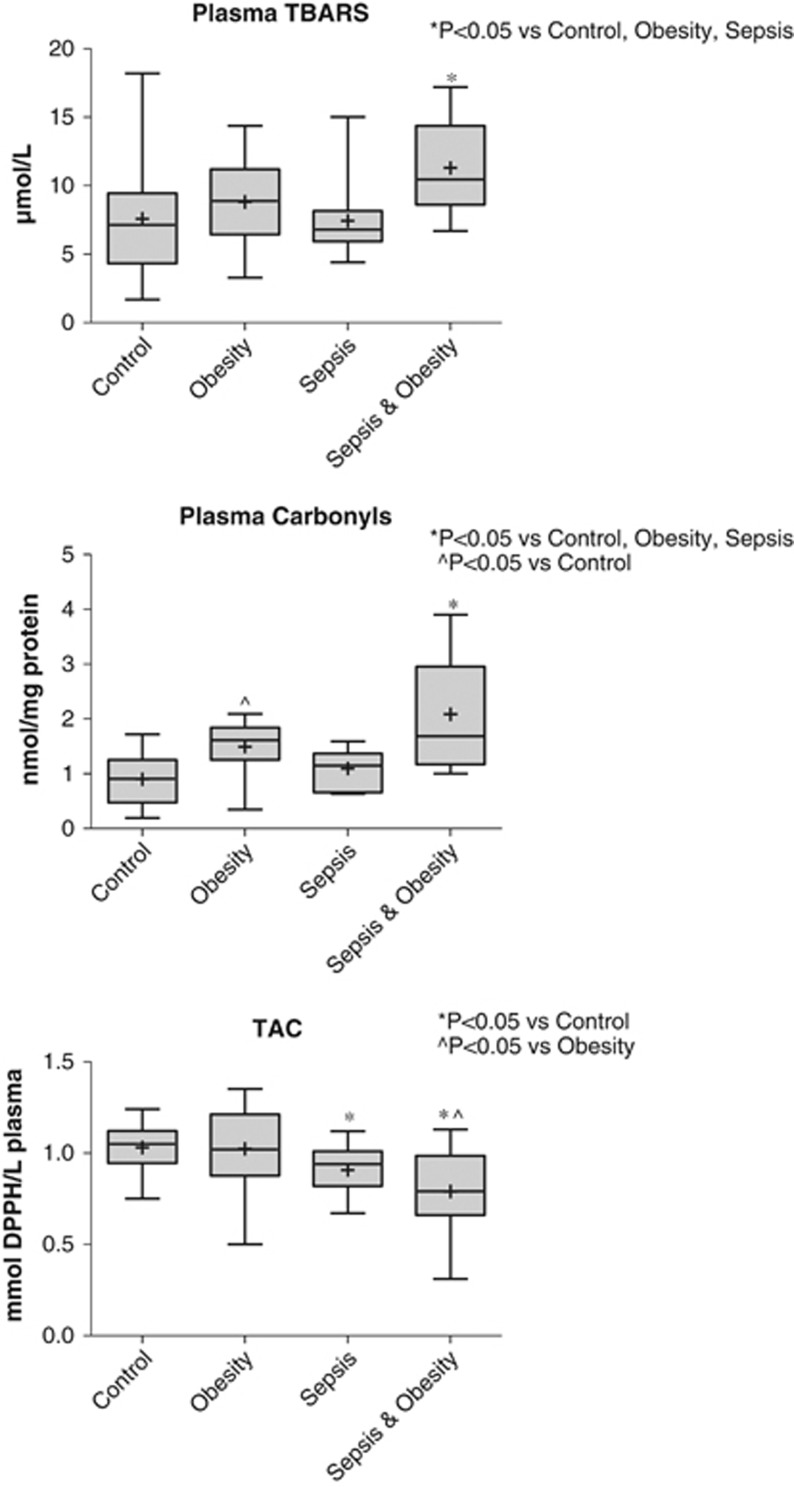
TBARS, protein carbonyls and TAC plasma levels of the four study groups (controls *n*=26, obesity *n*=27, sepsis *n*=27 and sepsis & obesity *n*=26).

The results of oxidative stress parameters' evaluation in plasma using TBARS, protein carbonyls and the TAC are shown in Figure 5.

Plasma TBARS levels were significantly higher in the sepsis & obesity group compared with the sepsis (*P*<0.001), obesity (*P*<0.05) and control (*P*<0.001) groups. Protein carbonyls in plasma were significantly higher in the sepsis & obesity group compared with the sepsis (*P*<0.001), obesity (*P*<0.05) and control (*P*<0.001) groups. In addition, the obesity group had significantly elevated levels of protein carbonyls compared with the control group (*P*<0.05).

The TAC levels of the sepsis & obesity group were significantly lower compared with the obesity group and the control group (*P*<0.001). Furthermore, the sepsis group had significantly lower TAC levels compared with the control group (*P*<0.05).

## Discussion

The concomitant presence of sepsis and obesity has proven to be an important cause of hospital admissions and mortality in the developed world.^1,12^ As a result, numerous clinical studies have attempted to investigate the potential effects of obesity on the outcome of septic patients with controversial results up to now.^18,36,37,38^ It is well established that obesity is associated with chronic low-grade inflammation characterized by abnormal cytokine production, increased acute phase reactants and activation of inflammatory signaling pathways.^39,40^ These changes promote important systemic effects that contribute to insulin resistance, dysmetabolism, cardiovascular diseases^41,42^ and compromise the patient's adaptive response to different etiologies of critical illness.^43,9^ However, the effect of obesity and accumulated adipose tissue on the immune response of septic patients in particular has not been adequately investigated. To the best of our knowledge, this is the first study attempting to investigate this issue by studying the potential differences in the adipose tissue and systemic immunologic response among obese septic and nonseptic patients, and nonobese septic and nonseptic patients.

Regarding the potential alterations in the adipose tissue macrophages number in the studied groups, our findings are in agreement with previously published reports on the effect of sepsis in adipose tissue macrophages.^23,24^ Specifically, septic patients demonstrated an increase in total macrophages as well as in their M1 and M2 subtypes, only in VAT and irrespective of BMI. Consequently, the increased immune response of septic patients could be mediated by the visceral component of adipose tissue, which has been increasingly recognized as an endocrine organ.^44^ However, the coexistence of excess of adipose tissue in the patients of the obesity groups does not seem to further exaggerate the number of total and M2 subtype macrophages compared with the nonobese groups. An interesting finding of our study is that the M2 macrophages predominate in the VAT of septic patients (obese and nonobese), which is in agreement with the proposed theory that critical illness provokes the accumulation of M2 macrophages in adipose tissue.^45^ In contrast to previously published reports,^25,46,47^ we found no increase in the concentrations of the macrophages in the VAT of obese individuals and additionally showed a prevalence of M2 macrophages, instead of M1, in the adipose tissue of the obese groups. A possible explanation for this unexpected finding was derived from the cell markers used for the immunohistochemistry detection of macrophages. Although CD68 is a specific marker for monocytes and macrophages, both CD206 and CD80 are known to be expressed, apart from macrophages, in other cells, like dendritic cells. Probably, the ostensibly increased number of CD206+ cells can be attributed to a standard error caused by the miscounting of other cells as macrophages in all study groups. Another explanation emanates from a currently proposed theory according to which, the majority of differences in the expression of a surface molecule in a given macrophage population are quantitative^26^ and studies have shown that adipose tissue macrophages can simultaneously express both M1 and M2 phenotypes.^48^ Analytical problems could have rendered macrophages with only minor expression of the CD206 marker immunohistochemically positive.

It has been already proposed that the expression of TNFα mRNA in adipose tissue is significantly elevated in obese individuals compared with lean controls.^49,50^ Our results, considering the TNFα mRNA levels in VAT, indicate that this phenomenon extends to septic patients. At the same time, our findings show that obesity does not increase the M1 proinflammatory macrophages in VAT, which are considered the main source of TNFα production in VAT. Consequently, the question raised is what could be the source of the increased TNFα production in the VAT of obese septic patients. A reasonable explanation, based on the findings of previous studies,^51,52^ is that TNFα in VAT of obese septic patients is produced by adipocytes. Nevertheless, this increase in TNFα mRNA levels in VAT is not reflected in the serum where increased TNFα levels in sepsis were not further influenced by obesity. This finding indicates that TNFα produced by VAT acts primarily in an autocrine/paracrine fashion, with only a small part of it entering the circulation.

In the present study, for the widest assessment of the oxidative status, we used three oxidative stress markers: (i) TBARS, a widely accepted marker of lipid peroxidation; (ii) protein carbonyls, a marker of protein oxidation; and (iii) TAC, an index of antioxidant defense. Sepsis has been shown to promote the systemic spread of oxidative stress, which additionally has been interrelated with the severity of septic syndrome.^29^ According to our results, sepsis reduced the plasma antioxidant defense without affecting the other two markers of lipid and protein peroxidation. Depletion of antioxidant defenses in septic patients has been previously reported repeatedly,^53,54^ whereas the lack of concomitant demonstration of increased lipid and protein oxidation in the plasma of septic patients, in the present study, may be explained by the potential increase of antioxidant substances like uric acid and bilirubin due to sepsis-induced organ dysfunction.^55,31^ In addition, the present study demonstrated that obesity increases protein oxidation. Potential mechanisms underlying the obesity-associated increase in oxidative stress are hyperglycemia, increased muscle activity to carry excessive weight, elevated tissue lipid levels and low-grade endotoxemia due to gut barrier dysfunction.^28,56^ Despite the observed inconsistency of oxidative stress results in the septic group and the obese group, the concomitant presence of sepsis and obesity induced an undoubted increase of oxidative stress, as assessed by all of the three markers used. This finding is indicative of a significant exacerbation of the oxidative stress process in obese septic patients, possibly induced by a combination of the promoting factors that are associated with each underlying condition *per se*. The presence of high oxidative stress in the systemic circulation of obese septic patients might further reflect the potential for systemic injurious structural and/or functional effects in multiple organs.

A limitation of this study is that it involves both surgical and medical patients. This is part of the study's protocol because of the anticipated difficulty to enroll surgical patients that fulfill both the inclusion and exclusion criteria in all the four study groups and our effort to increase the number of samples for the oxidative stress techniques. Care was taken so that each one of the four study groups has almost the same number of surgical and medical patients. Therefore, the study's results are considered to be comparable between the four study groups. Another limitation of the study is associated with the VAT biopsies obtained from patients with abdominal sepsis. It would have been ideal to take VAT from patients with an extra-abdominal source of sepsis. As this scenario is forbidden owing to obvious ethical reasons, we carefully selected patients with localized intra-abdominal infection, without peritonitis. In addition, VAT biopsies were obtained away from the site of inflammation and therefore, it can be speculated that the number of macrophages in VAT reflect the generalized immune reaction rather than a local phenomenon. Owing to the above-mentioned restriction, considering the localized intra-abdominal infection, the study's patients in total were selected to have minor septic insults in order to conclude in comparable results. This limitation could have overshadowed more prominent differences in the number of macrophages and the TNFα levels that might be revealed in severely septic patients.

In conclusion, the present study demonstrates that underlying obesity in the presence of sepsis is associated with increased proinflammatory cytokine production by the VAT, which seems to act locally, as well as with exaggerated systemic oxidative stress. Future studies focused on the clinical impact, in terms of morbidity and mortality, of these or additional pathophysiological alterations in obese septic patients might enhance our understanding on how the epidemic of obesity might affect patients' outcome in the case of superimposed sepsis.

## Figures and Tables

**Table 1:** Demographic characteristics of patients enrolled in the four study groups

*Study groups*	*Number of patients (gender)*	*Age (years) (mean±s.d.)*	*BMI (kg m*^*−2*^*) (median (25–75%.))*
Control	*n*=26 (11 F, 15 M)	54±18	24.7 (23.9–27.5)
Obesity	*n*=27 (20 F, 7 M)	58±17	34.2 (31.2–42.0)
Sepsis	*n*=27 (9 F, 18 M)	62±26	24.8 (21.7–28.0)
Sepsis & obesity	*n*=26 (11 F, 15 M)	62±20	32.0 (30.3–34.5)

Abbreviations: BMI, body mass index; F, female; M, male.

Control group, nonseptic and nonobese; obesity group, nonseptic and obese; sepsis group, septic and nonobese; sepsis & obesity group, septic and obese.
